# Impact of Aspiration Pneumonia on the Clinical Course of Progressive Supranuclear Palsy: A Retrospective Cohort Study

**DOI:** 10.1371/journal.pone.0135823

**Published:** 2015-08-13

**Authors:** Satoshi Tomita, Tomoko Oeda, Atsushi Umemura, Masayuki Kohsaka, Kwiyoung Park, Kenji Yamamoto, Hiroshi Sugiyama, Chiaki Mori, Kimiko Inoue, Harutoshi Fujimura, Hideyuki Sawada

**Affiliations:** 1 Clinical Research Center, National Regional Center for Neurological Disorders and Utano National Hospital, Kyoto, Japan; 2 Department of Neurology, National Regional Center for Neurological Disorders and Utano National Hospital, Kyoto, Japan; 3 Department of Neurology, Toneyama National Hospital, Osaka, Japan; Hospital General Dr. Manuel Gea González, MEXICO

## Abstract

**Introduction:**

Although aspiration pneumonia is the most common complication of progressive supranuclear palsy (PSP), the clinical impact of aspiration pneumonia on disease course and survival has not been fully estimated. Thus, we retrospectively analyzed the prognostic factors and clinical consequences of pneumonia in PSP.

**Methods:**

The clinical course of patients with aspiration pneumonia was surveyed. The association between baseline clinical features (2 years from disease onset) and latency to the initial development of pneumonia was investigated using survival time and Cox regression analyses.

**Results:**

Ninety patients with a clinical diagnosis of PSP were observed for 5.1±3.8 years (mean±SD), and 22 had aspiration pneumonia. Subsequently, 20 patients (91%) had to discontinue oral feeding entirely and 13 (59%) died, whereas, of 68 patients without pneumonia, only three patients (4%) died. Time to initial development of pneumonia was strongly correlated with survival time (Spearman R = 0.92, *P*<0.001), with a mean latency of 2.3 years to death. Among baseline clinical features, early fall episodes and cognitive decline were significant predictors of pneumonia (*P* = 0.001 and *P*<0.001, respectively, log rank test). Cox regression analysis demonstrated that early fall episodes (adjusted hazard ratio: 3.9, 95% confidence interval: 1.2–12.5, *P* = 0.03) and cognitive decline (adjusted hazard ratio: 5.2, 95% confidence interval: 1.4–19.3, *P* = 0.02) independently predicted pneumonia. By contrast, dysphagia was not associated with pneumonia (*P* = 0.2, log rank test).

**Conclusion:**

Initial development of pneumonia indicates an unfavorable clinical course and predicts survival time (mean survival time 2.3 years). Patients with early falls and cognitive decline were at high risk of early development of pneumonia.

## Introduction

Progressive supranuclear palsy (PSP) is a neurodegenerative disease presenting with symptomatic parkinsonism including bradykinesia, muscular rigidity, and postural reflex disturbance. In addition, most patients with PSP suffer from dementia and dysphagia. The most common cause of death in PSP is pneumonia [1], which occurs subsequent to silent aspiration resulting from pre-existing dysphagia [2]. Dysphagia is a well-recognized complication of PSP [3], occurring in up to 80% of patients [4]. Both the time to development of dysphagia and that from dysphagia to death are shorter in PSP than in Parkinson disease (PD) [5]. In addition, PSP patients with early development of dysphagia have a short survival time [4] because repeated aspiration pneumonia can be fatal. Therefore, physicians attempt to prevent aspiration pneumonia by medication or palliative treatment, including adjusting food consistency, feeding techniques, and gastrostomy tube feeding [6]. The timely application of these treatments is important to maintain the quality of life of patients, and identification of predictive factors of early development of aspiration pneumonia is required to prevent serious consequences of dysphagia in PSP [7]. However, factors that reliably predict the early development of pneumonia and subsequent clinical courses have not been fully elucidated. PSP is clinically heterogeneous, especially in the early stages. Although several studies have reported early clinical features associated with prognosis of PSP [8–11], studies focusing on the impact of pneumonia on the clinical course of PSP are lacking. Here, we investigated the clinical consequences of pneumonia and the association between early clinical features and latency to the initial development of pneumonia, using a survival time analysis in a retrospective cohort.

## Materials and Methods

### Study Design

To identify factors associated with the early development of pneumonia in PSP, a retrospective cohort study was designed and a survival time analysis was adopted. The main outcome measure was the time from the start of study observation to the initial development of pneumonia. Observation began 2 years from clinical onset (2 years from an initial neurological symptom), and clinical symptoms and signs of PSP during these 2 years were regarded as baseline features.

### Patients

We studied consecutive patients with parkinsonism who were followed in the Utano National Hospital Parkinson Disease Center between November 2006 and December 2014. Patients were admitted to our hospital and examined by two or more neurologists with expertise in movement disorders. Detailed clinical evaluations were undertaken to determine whether they fulfilled clinical criteria for a probable or possible diagnosis of PSP, according to the PSP criteria of the National Institute of Neurological Disorders and Stroke and the Society for PSP (NINDS-SPSP) [8]. Patients who fulfilled PD criteria in the UK Brain Bank Clinical Diagnostic Criteria (Steps 1 and 2) [12] were excluded. To exclude a possible diagnosis of multiple system atrophy or other diseases manifesting as parkinsonism, brain magnetic resonance imaging was performed. To exclude dopa-responsive parkinsonism represented by PD, the response to an adequate dose of levodopa was confirmed in all cases. Patients observed for <2 years from the initial symptoms and signs were excluded because the start of the study observation was set at 2 years from disease onset. Patients with pseudobulbar palsy due to cerebrovascular diseases were also excluded. Immunocompromised patients (e.g., by hematological disorders) were also excluded because they are prone to infections including pneumonia.

### Data Collection

Clinical factors such as early fall episodes, cognitive decline, supranuclear gaze palsy, tremor, and others were collected from medical records within 2 years from disease onset. The data were recorded on data sheets designed for this study. Structured interviews were performed with patients or their families at the hospital, or on the telephone to complete the data sheets. The following clinical data were also collected: age at disease onset, observation period, initial symptom at disease onset, time from disease onset to dysphagia, and cause of death. Smoking history was also noted because smoking is significantly associated with the development of aspiration pneumonia [13]. Patients who had smoked at disease onset were included in a smoker group.

### Main Outcome Measure

The time from the start of the study observation to the first episode of pneumonia was the main outcome measure. A diagnosis of pneumonia was made according to the following criteria: clinical signs and symptoms, white blood cell count ≥10,000/μL or proportion of neutrophils ≥80%, serum C-reactive protein level ≥60 mg/L, fever (body temperature >37°C), and new infiltrates or consolidations on chest radiography (X-ray or computed tomography). Detailed data were collected for patients who had been treated for pneumonia in another hospital or by a primary care physician to determine whether the above criteria for pneumonia had been met. Patients who died before developing pneumonia were classified as having an alternative outcome.

### Possible Predictive Factors

To identify predictive clinical factors for pneumonia, a comprehensive review of the symptoms and signs important in making a diagnosis of PSP was performed. We collected data on the following clinical features and phenotypes at the start of the study observation period: fall episodes, cognitive decline, bradykinesia, dysarthria, dysphagia, tremor, asymmetric onset of extrapyramidal signs, postural reflex disturbance, extra axial-dystonia, supranuclear gaze palsy, abnormal saccade or pursuit eye movements, and ever having a response to levodopa. These features have previously been used by Williams et al. to characterize clinical phenotypes of PSP [14]. The definitions of these symptoms and signs are shown in S1 Table. According to the definitions [14] proposed by Williams et al., patients were classified into Richardson’s syndrome (RS) or PSP-parkinsonism (PSP-P) phenotypes on the basis of baseline clinical features. When clinical features supporting these two phenotypes were equal or data about baseline features were incomplete, patients were grouped as unclassifiable.

### Statistical Methods

The incidence of pneumonia was estimated as the number of patients developing pneumonia divided by the corresponding person-years at risk. The relationship (A) between latency to dysphagia and total survival time and (B) between latency to first pneumonia and total survival time was examined using Spearman’s rank correlation coefficient (in deceased cases with experience of pneumonia). The risk of early development of pneumonia associated with each predictive factor was investigated by survival time analysis. Thereafter, the risk associated with significant predictive factors as detected by survival time analysis was evaluated using hazard ratios (HRs) by Cox regression analysis, adjusted by sex and age at disease onset. A previous large study of pathologically confirmed PSP patients has indicated that older age at onset and male sex are associated with poor life prognosis [10].

Analyses were performed using PASW Statistics version 18 (SPSS Inc., Chicago, IL, USA). Results are expressed as mean ± SD, and statistical significance was defined as *P*<0.05.

### Ethics

This study was approved by the Bioethics Committee of Utano National Hospital (registry number: 25–21) and the protocol was consistent with the principles of the Declaration of Helsinki. The Bioethics committee waved the need for informed consent due to the retrospective nature of the study and anonymity of the collected data, according to the Ethical Guideline for Medical and Health Research Involving Human Subjects from Ministry of Health, Labour and Welfare, Japan.

## Results

According to the NINDS-SPSP criteria, 100 patients were diagnosed as probable or possible PSP. All patients showed supranuclear gaze palsy. The response to levodopa was reviewed in detail in all patients, and none showed a moderate to excellent response, but dopaminergic therapy had a modest beneficial effect in 20 patients. Ten patients were excluded for the following reasons. Eight patients had disease duration <2 years, one had a history of cerebral infarction causing bulbar dysfunction, and one had comorbid multiple myeloma. As a result, 90 patients who were diagnosed with PSP were eligible and all were enrolled into the study.

Anonymized case data set of the study was registered at Dryad Data Repository site.

### Demographic Characteristics of the Patients

The demographic characteristics of the 32 female and 58 male patients are shown in S2 Table. The mean age at PSP onset was 68.6 years and the mean disease duration at study enrollment was 7.1 years. The mean study observation period was 5.1 years. During the observation period, 22 patients developed pneumonia, and the incidence was 55 (95% confidence interval [CI]: 32–77) per 1,000 person-years. Initial symptoms at disease onset are shown in S2 Table. The most frequent initial symptom was fall, which occurred in 39% of patients. During the observation period, 16 patients died and the mean disease duration of these patients was 9.0 years. Ten (71%) of 14 patients in whom the cause of death was known died of complications associated with severe dysphagia; five of them died of recurrent pneumonia, three died of sepsis related to the total parenteral nutrition catheter, and two died of suffocation.

### Clinical Course with Pneumonia

**Fig 1:**
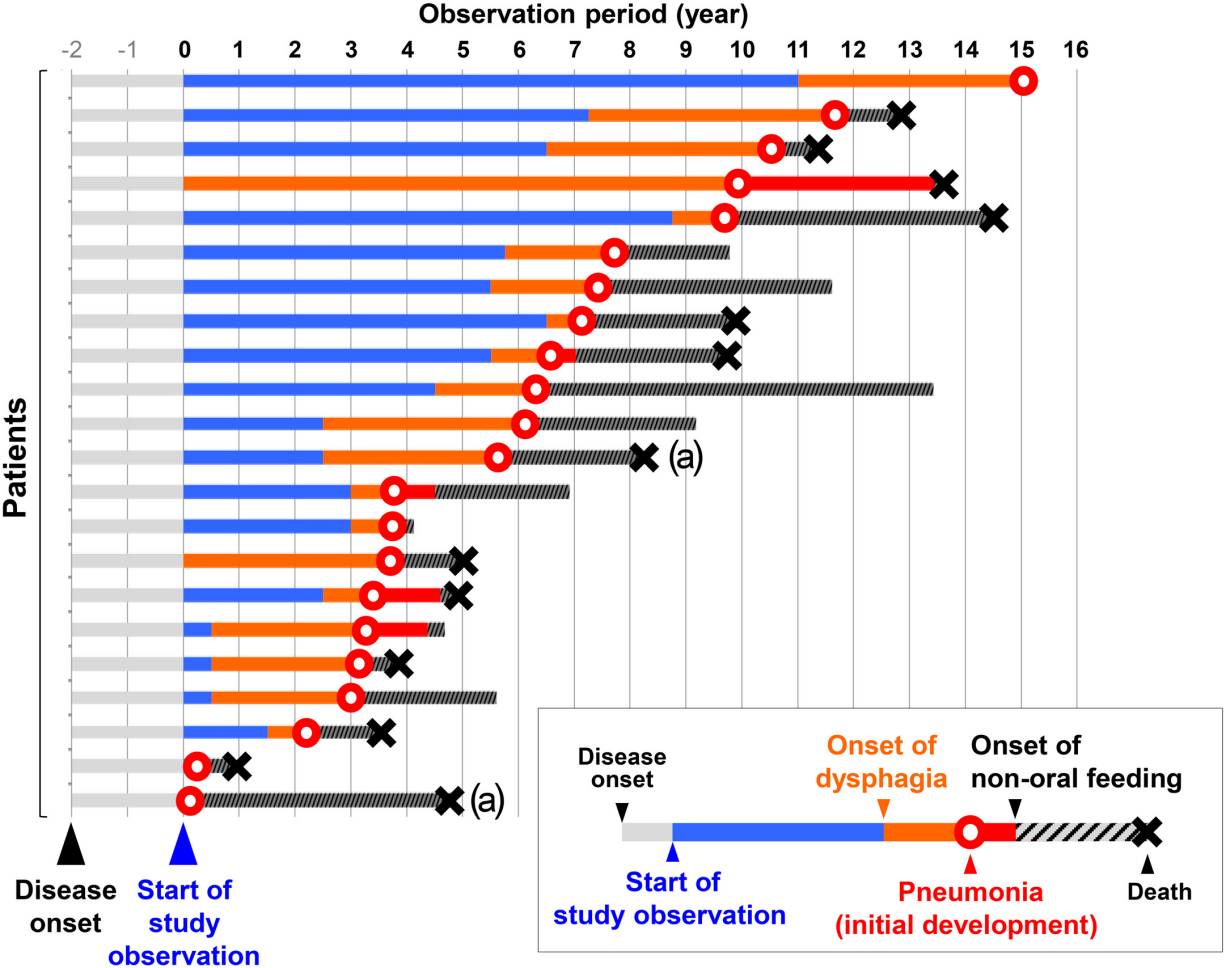
Clinical courses of 22 patients with experience of pneumonia. Pneumonia developed in 22 patients and 13 patients died in the observation period. ^a^Pathologically confirmed cases of progressive supranuclear palsy.

Fig 1 shows the chronological clinical course of 22 patients who had experienced pneumonia. In the study period, 13 (59%) of these patients died, and the cause of death was pneumonia (n = 5), sepsis (n = 2), suffocation (n = 1), and other (n = 5). Two of the deceased patients were investigated pathologically, and the diagnosis of PSP was confirmed according to neuropathological diagnostic criteria [15]. Neuronal fibrillary tangles and glial inclusions (tufted astrocytes) were seen in the basal ganglia and brainstem, which is a typical distribution in PSP.

Of 22 patients with pneumonia, radiographic examinations by chest CT or X-ray showed new dorsal infiltration or consolidation consistent with aspiration pneumonia in 21 patients, but detailed radiological data were not obtained in one patient with pneumonia treated in another hospital. All 22 patients suffered from moderate to severe dysphagia before the development of pneumonia. Time to initial development of pneumonia from study enrollment was <4 years in 10 patients; however, it ranged from 0 to 15 years. After the initial development of pneumonia, 20 patients (91%) required non-oral feeding (NOF), including nasogastric tube feeding, percutaneous gastrostomy (PEG) tube feeding, and total parenteral nutrition (Table 1). Owing to severe or persistent dysphagia, NOF was not discontinued in any of these patients during the observation period. Of 20 patients with NOF, 18 finally required PEG tube feeding, and two required total parenteral nutrition because of recurrent pneumonia under nasogastric tube feeding. Mean survival time after NOF was 2.0 years (range 0.2–4.8 years).

**Table 1:** Comparison of patients with and without the experience of aspiration pneumonia.

		Aspiration pneumonia		
	All (n = 90)	+ (n = 22)	- (n = 68)	P value
Total observation period, person-years	-	183.4	272.3	
Age of disease onset, years (mean ± SD)	68.6 ± 7.1	68.8 ± 7.7	68.5 ± 6.9	0.84^a^
Male, n (%)	58 (64)	16 (73)	42 (62)	0.45^b^
Smoking history, n (%)	19 (21)	5 (23)	14 (21)	1.00^b^
Latency^c^ to dysphagia, years (mean ± SD)	4.4 ± 2.7	6.0 ± 3.1	4.0 ± 2.4	0.04^a^
Latency^c^ to first pneumonia, years (mean ± SD)	-	7.9 ± 3.9	-	-
Cases with non-oral feeding, n (%)	21 (23)	20 (91)	1 (1)	<0.001^b^
Number of deceased cases, n (%)	16 (18)	13 (59)	3 (4)	<0.001^b^
**Clinical features during the first 2 years of disease**				
Fall episodes, n (%)	60 (67)	15 (68)	45 (66)	1.00^b^
Cognitive decline, n (%)	24 (27)	5 (23)	19 (28)	0.78^b^
Dysarthria, n (%)	42 (47)	9 (41)	33 (49)	0.63^b^
Dysphagia, n (%)	24 (27)	4 (18)	20 (29)	0.41^b^
Tremor, n (%)	18 (20)	7 (32)	11 (16)	0.13^b^
Asymmetric onset of extrapyramidal signs, n (%)	20 (23)	6 (29)	14 (21)	0.55^b^
Bradykinesia, n (%)	52 (78)	11 (79)	41 (77)	1.00^b^
Postural reflex disturbance, n (%)	52 (91)	10 (83)	42 (93)	0.28^b^
Extra axial-dystonia, n (%)	25 (50)	2 (20)	23 (57)	0.07^b^
Supranuclear gaze palsy, n (%)	35 (69)	9 (75)	26 (67)	0.73^b^
Abnormal saccade or pursuit eye movements, n (%)	34 (76)	4 (57)	30 (79)	0.34^b^
Response to levodopa ever, n (%)	21 (33)	7 (47)	14 (29)	0.23^b^
RS / PSP-P phenotype, n	48 / 13	10 / 5	38 / 8	0.28^b^

RS/PSP-P, Richardson syndrome / PSP-Parkinsonism

^a^Mann–Whitney test

^b^Fisher’s exact test

^c^Latency from disease onset

S1 Fig shows the correlation between survival time and latency to dysphagia and that between survival time and latency to first pneumonia in the 13 deceased patients with experience of pneumonia. Latency to dysphagia correlated with survival time (Spearman R = 0.60, *P* = 0.03), and latency to first pneumonia was strongly correlated with survival time (Spearman R = 0.92, *P*<0.0001). Mean life expectancy after initial development of pneumonia was 2.3 years (SD 1.4 years, range 0.7–4.8 years). The equation produced by a linear regression model of the relationship between total survival time (*y*) and latency to pneumonia (*x*) was *y* = 1.05 *x* + 1.9 (years). As expected, there was also a moderate correlation between latency to the onset of dysphagia and pneumonia in the 22 patients (Spearman R = 0.67, *P* = 0.01).

A comparison of demographic data and baseline clinical features (in the first 2 years of the disease) between patients with and without experience of pneumonia is shown in Table 1. Patients with pneumonia had to discontinue oral feeding and died more frequently than those without pneumonia. The most frequent baseline clinical feature was postural reflex disturbance, and the second most frequent was abnormal saccade or pursuit eye movements. The least frequent feature was tremor. Forty-eight patients were classified as RS and 13 as PSP-P. There were no significant differences in baseline clinical features and phenotypes between patients with and without pneumonia.

### Survival Time Analysis

**Fig 2:**
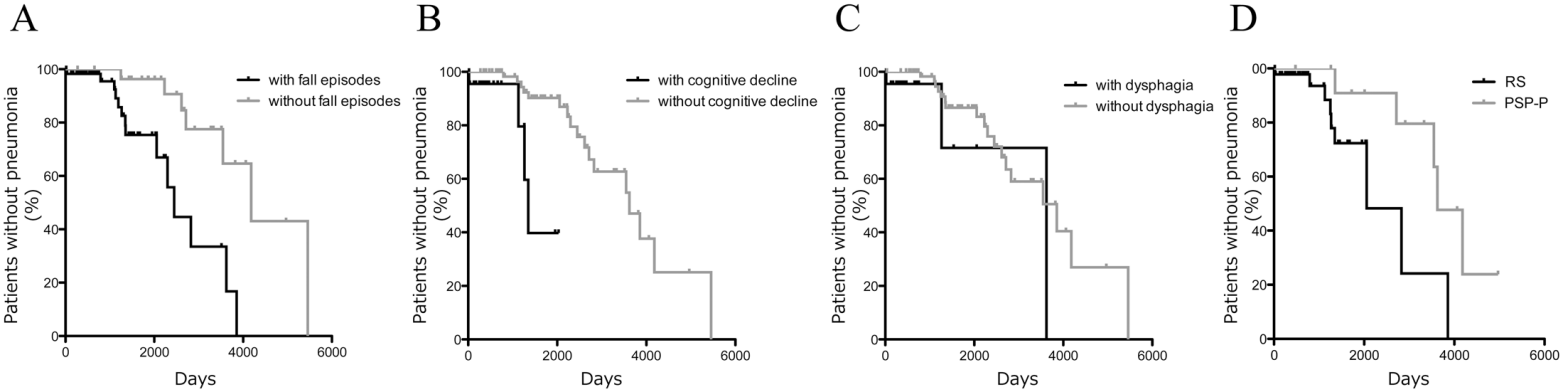
Survival analyses stratified by patients’ clinical features and phenotypes during initial 2 years of disease. Latency from the start of the study to the initial development of pneumonia, stratified by with or without (A) fall episodes (log rank *P* = 0.001), (B) cognitive decline (log rank *P*<0.001), (C) dysphagia (log rank *P* = 0.08), and (D) clinical phenotypes (RS and PSP-P; log rank *P* = 0.05).

Fig 2 shows the Kaplan–Meier curves of the cumulative proportion of patients without pneumonia stratified by baseline clinical features and phenotypes. Fall episodes (log rank test, *P* = 0.0006) and cognitive decline (*P* = 0.0025) were significant predictors of shorter latency to pneumonia; however, dysphagia was not significant (*P* = 0.2). The RS phenotype was a significant risk factor for early development of pneumonia (*P*<0.0001).

There was no significant difference in the pneumonia latency between patients stratified by dysarthria (*P* = 0.2), tremor (*P* = 0.9), asymmetric onset of extrapyramidal signs (*P* = 0.9), bradykinesia (*P* = 0.4), postural reflex disturbance (*P* = 0.7), extra axial-dystonia (*P* = 0.07), supranuclear gaze palsy (*P* = 0.08), abnormal saccade or pursuit eye movements (*P* = 0.4), or response to levodopa ever (*P* = 0.2).

Cox regression analysis indicated that cognitive decline at baseline was the most significant risk factor for pneumonia (adjusted HR: 5.2, 95% CI: 1.4–19.3, *P* = 0.02), and it was still the most significant risk factor in the model after adjustment for dysphagia as a baseline feature (adjusted HR: 5.2, 95% CI: 1.4–19.3, *P* = 0.01). Early fall episodes were also a significant risk factor of pneumonia (adjusted HR: 3.9, 95% CI: 1.2–12.5, *P* = 0.03; Table 2).

**Table 2:** Cox proportional hazards regression models for the predictive factors of early development of aspiration pneumonia.

	Prognostic variables	Adjusted hazard ratio	95% confidence interval	*P* value
Model 1	**Cognitive decline**	**5.2**	**1.4–19.3**	**0.015**
	**Fall episodes**	**3.9**	**1.2–12.5**	**0.023**
Model 2	**Cognitive decline**	**6.0**	**1.4–19.3**	**0.014**
	**Fall episodes**	**4.0**	**1.2–12.9**	**0.021**
	Dysphagia	0.8	0.2–3.2	0.756

Model 1, HR adjusted by age and sex; Model 2, HR adjusted by age, sex, and dysphagia (yes/no) during the first 2 years of the disease.

## Discussion

The current study demonstrated that latency to the initial development of pneumonia was strongly correlated with survival time, and early clinical symptoms could predict shorter latency to pneumonia in PSP. After initial development of pneumonia, all patients required inpatient care and most had to discontinue oral feeding as a result of severe dysphagia. There was only a 2.3-year latency from the initial development of pneumonia to death. This report provides a reminder that the initial development of pneumonia is a noteworthy event in PSP because it has a serious negative affect on both quality of life and survival.

In this study, early fall episodes and cognitive decline predicted a shorter latency to pneumonia, and these features were independent significant risk factors in Cox regression analysis. In a series of recent studies, early clinical features such as fall [8, 9], cognitive decline [9, 11], dysphagia [8, 11], dysarthria, and diplopia [8] were associated with shorter survival time, in addition to the established risk factors, age at onset [9–11, 16] and male sex [10, 16]. The clinical phenotype of RS in which fall and cognitive decline are prominent is also a significant risk factor for shorter survival [10, 11]. In our cohort, early fall episodes, cognitive decline, and RS phenotype were confirmed as significant risk factors for shorter survival (S2 Fig). Therefore, our study results suggested that predictors of a shorter latency to pneumonia and shorter survival time were relevant, and the initial development of pneumonia could be used as a reliable surrogate marker of an increased risk of mortality in PSP.

The most common cause of death in PSP is aspiration pneumonia [4]. In our study cohort, dysphagia-related death, which included aspiration pneumonia, sepsis related to total parenteral nutrition catheter, and suffocation, accounted for 91% of deaths with a known cause. Intriguingly, dysphagia as a baseline feature was rarely associated with early development of pneumonia. Initially, dysphagia was regarded as an early sign of PSP and was listed as a supportive feature in diagnostic criteria [2, 17]. However, recent studies demonstrated that dysphagia is a less frequent feature in the early stage [14] but becomes more frequent in the mid-to-late stage [18–20]. Mean latency to dysphagia was 3.4–4.7 years in previous studies [5, 20–23] and 4.4 years in our study cohort, including RS patients as well as patients with PSP-P and pure akinesia with gait freezing (PAGF). The last two phenotypes have a slowly progressive clinical course and generally mild tau deposition [24–26]. Latency to dysphagia of RS patients (3.1±1.9 years) was significantly shorter than that of PSP-P patients (6.6±3.5 years; S3 Table).

PSP patients are more accurate in expressing their swallowing difficulties than PD patients, and a swallowing questionnaire is useful method to predict the swallowing disturbance in PSP [2]. To improve survival, an appropriate and timely swallowing evaluation and intervention for silent aspiration resulting from dysphagia may be important in pre-pneumonia phase. Actually, our patients were questioned about their swallowing problems at every interview, and then instructed to use a fluid thickener and avoid dry and sticky food prior to developing pneumonia. Although the results of the current study failed to show the association of early-onset dysphagia and pneumonia development, it does not exclude the possibility that the earlier detection of dysphagia and such instruction about food consistency does not delay the development of aspiration pneumonia.

While some patients with PSP show levodopa responsiveness and mild improvement of dysphagia [27, 28], management of dysphagia in the later stages of PSP is more difficult. In our study, most patients with NOF finally required PEG tube feeding. In spite of palliative treatments including adjusting food consistency and feeding techniques, a PEG tube was placed a few months after the initial development of pneumonia in most of the patients in the current study. Mean disease duration of the 16 deceased cases (including 10 patients with a PEG tube) was 9.0 years (S3 Table), which is longer than that previously reported (6.6–8.0 years) [4, 10, 11]. PEG tube feeding was expected to decrease the risk of pneumonia and prolong survival. In our cohort, it was unclear whether PEG placement prolonged survival. Further prospective study is required to confirm the life advantage effect of PEG placement in PSP.

The current study had several limitations. They include selection bias arising from a single-center population and a retrospective study design. In addition, the application of a clinical diagnosis of PSP remains a major methodological limitation. In the current study data were not collected according to predefined schedule and several data were missing; however, they were collected at least monthly in most patients. The neurological signs and symptoms were evaluated by expert neurologists. Therefore, the completeness and accuracy of the data were limited but enough to draw the conclusions.

### Conclusions

In summary, better knowledge of the early symptoms of PSP, especially fall episodes and cognitive decline, will be useful for the prediction and prevention of the development of pneumonia, and will support a better clinical course. We found that the initial development of pneumonia predicted a short survival time and should be recognized as one of the significant clinical milestones in the middle or late stage of PSP.

## Supporting Information

S1 Fig Correlation between survival time and latency to dysphagia or initial pneumonia in 13 deceased patients. The starting point (zero) indicates the start of study observation (2 years from disease onset). A 95% CI is indicated in gray. (A, Spearman R = 0.60, *P* = 0.03; B, Spearman R = 0.92, *P*<0.001).
(TIFF)

S2 Fig Survival analyses stratified by patients’ clinical features and phenotypes during first 2 years of disease. (A–D) Survival time stratified by with or without (A) fall episodes (log rank *P* = 0.015), (B) cognitive decline (log rank *P*<0.001), (C) dysphagia (log rank *P* = 0.018), and (D) clinical phenotypes (RS and PSP-P; log rank *P* = 0.002).
(TIFF)

S1 Table Definitions of baseline clinical symptoms and signs used in the study. (DOCX)

S2 Table Demographic characteristics of the 90 study patients. (DOCX)

S3 Table Comparison of patients with RS and PSP-P. (DOCX)
