# Supplementary figures and images for: Impact of Aspiration Pneumonia on the Clinical Course of Progressive Supranuclear Palsy: A Retrospective Cohort Study
Source: PLoS One. 2015 Aug 13;10(8):e0135823. doi: 10.1371/journal.pone.0135823 (PMC4536232; doi:10.1371/journal.pone.0135823)

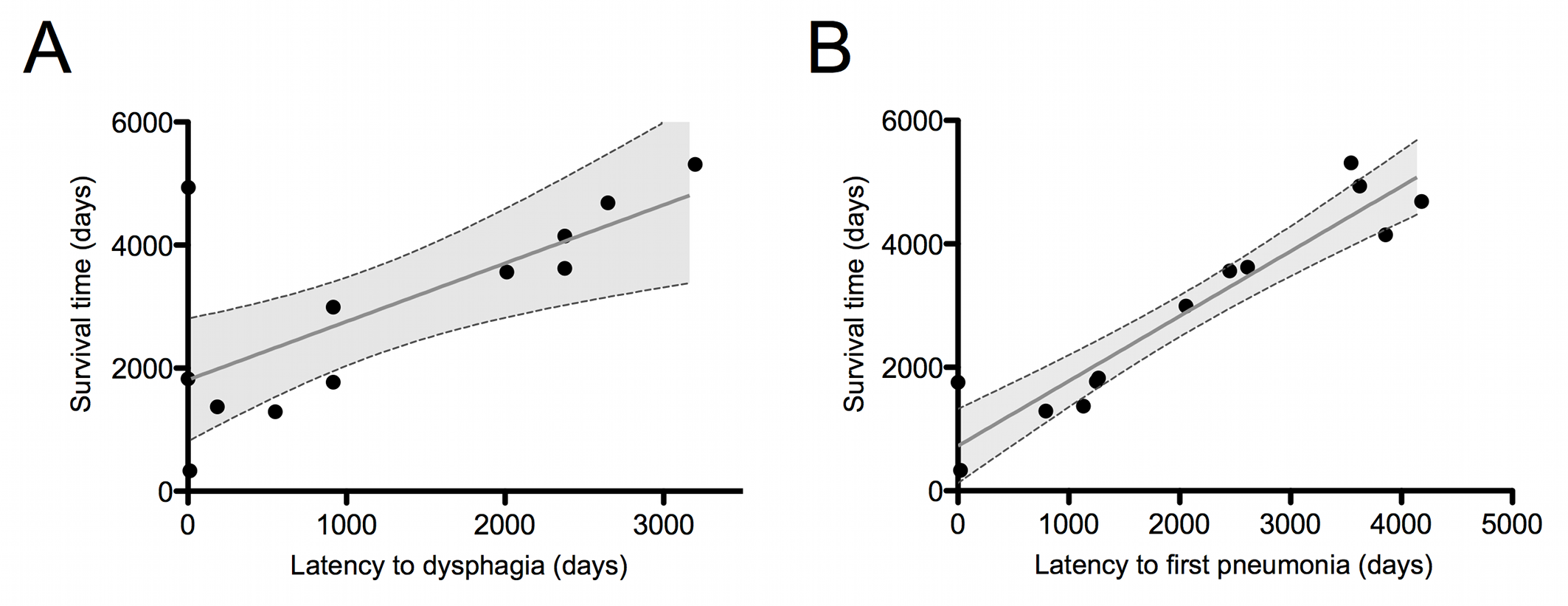

Supplement: S1 Fig — The starting point (zero) indicates the start of study observation (2 years from disease onset). A 95% CI is indicated in gray. (A, Spearman R = 0.60, P = 0.03; B, Spearman R = 0.92, P<0.001). (TIFF) [file pone.0135823.s001.tiff]

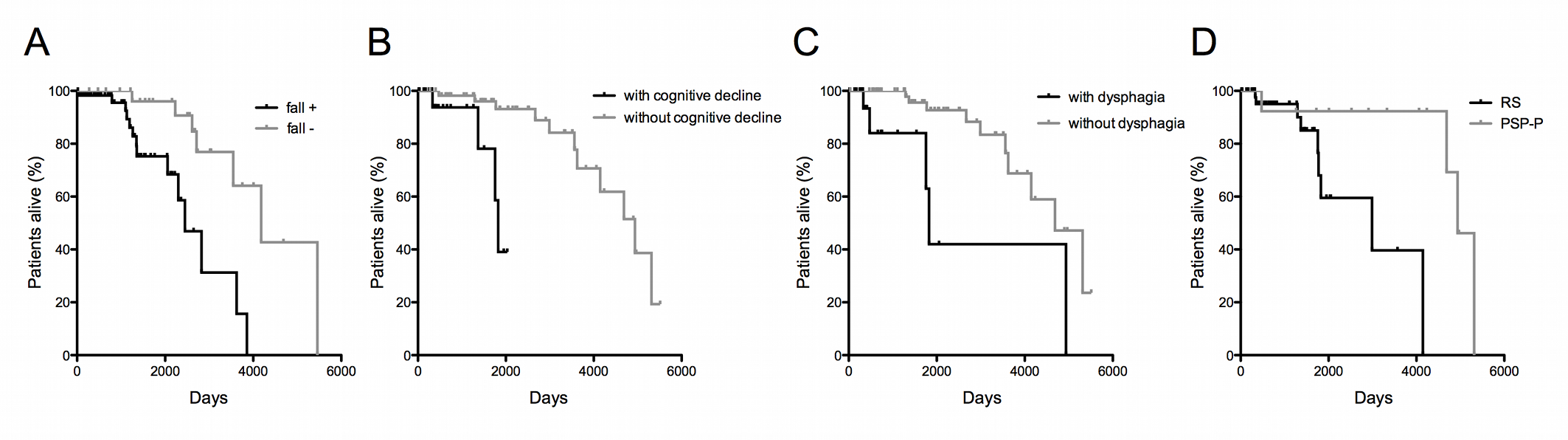

Supplement: S2 Fig — (A–D) Survival time stratified by with or without (A) fall episodes (log rank P = 0.015), (B) cognitive decline (log rank P<0.001), (C) dysphagia (log rank P = 0.018), and (D) clinical phenotypes (RS and PSP-P; log rank P = 0.002). (TIFF) [file pone.0135823.s002.tiff]
