# Supplementary material for: Impact of Aspiration Pneumonia on the Clinical Course of Progressive Supranuclear Palsy: A Retrospective Cohort Study
Source: PLoS One. 2015 Aug 13;10(8):e0135823. doi: 10.1371/journal.pone.0135823 (PMC4536232; doi:10.1371/journal.pone.0135823)
Supplement: S1 Table — (DOCX) [file pone.0135823.s003.docx]

**S1 Table. Definitions of baseline clinical symptoms and signs used in the study**

| **Feature** | **Definition** |
| --- | --- |
| Fall episodes | Description of fall episodes regardless of the cause. |
| Cognitive decline | Patient’s, caregivers’ or clinician’s perception of any present cognitive decline. This included descriptions of episodes of bradyphrenia, changes in personality, and a slowing of thought processes. If patients underwent neuropsychological tests (e.g., the Mini-Mental State Examination (MMSE)), the results of such tests were referenced. When a combination of cognitive decline, positive neuropsychological tests, and DSM-IV^a^ definition of dementia could not be obtained, the patient was treated as a missing value. |
| Bradykinesia | Presence of any mention of bradykinesia or motor slowing. |
| Dysarthria | Description of any alteration in speech quality compared with speech prior to disease onset. |
| Dysphagia | Description of swallowing abnormalities, including the documentation of patient’s subjective and caregivers’ objective impressions. |
| Tremor | Description of any type of tremor. |
| Asymmetric onset of extrapyramidal signs | If there was a difference in the motor signs between the left and the right sides of the body, asymmetry was recorded as being present. This included asymmetry of tremor, rigidity, bradykinesia, or functional decline. |
| Postural reflex disturbances | Postural reflex disturbances |
| Extra axial-dystonia | Presence of dystonia in any body part apart of the trunk and neck. |
| Supranuclear gaze palsy | Medical recording of a restricted range of eye movement in vertical directions. |
| Abnormal saccade or pursuit eye movements | Medical recording of abnormal saccadic or smooth pursuit eye movements. |
| Ever having a response to levodopa | Patient and clinician’s interpretation of improvement was assessed from case notes, and in some cases, according to improvement in the motor scores of United Parkinson Disease Rating Scale (UPDRS part III) after levodopa administration. |

^a^Diagnostic and Statistical Manual of Mental Disorders, Fourth Edition.
