# Supplementary material for: Impact of Aspiration Pneumonia on the Clinical Course of Progressive Supranuclear Palsy: A Retrospective Cohort Study
Source: PLoS One. 2015 Aug 13;10(8):e0135823. doi: 10.1371/journal.pone.0135823 (PMC4536232; doi:10.1371/journal.pone.0135823)
Supplement: S2 Table — (DOCX) [file pone.0135823.s004.docx]

**S2 Table. Demographic characteristics of the 90 study patients**

| Number of patients | 90 |
| --- | --- |
| Age of disease onset, years (mean ± SD) | 68.6 ± 7.1 |
| Observation period^a^, years (mean ± SD) | 5.1 ± 3.8 |
| Male, n (%) | 58 (64) |
| Probable / Possible progressive supranuclear palsy, n (%) | 55 (61) / 35 (39) |
| Incidence of pneumonia during observation period, n (%) | 22 (24) |
| Number of deceased cases, n (%) | 16 (18) |
| Total disease duration of deceased cases, years (mean ± SD) | 9.0 ± 4.6 |
| Initial symptom at disease onset | n (%) |
| Fall | 35 (39) |
| Bradykinesia | 14 (16) |
| Gait disturbance | 15 (17) |
| Hand clumsiness | 8 (9) |
| Tremor | 8 (9) |
| Speech disturbance | 5 (6) |
| Difficulty of eye-lid opening | 4 (4) |
| Hypomimia | 1 (1) |
| Cause of death in deceased cases | n (%) |
| Recurrent pneumonia | 5 (31) |
| Sepsis (related to total parenteral nutrition catheter) | 3 (19) |
| Suffocation | 2 (13) |
| Cancer | 2 (13) |
| Acute cholecystitis | 1 (6) |
| Sudden death | 1 (6) |
| Unknown | 2 (13) |

^a^From 2 years of the disease to the end of the observation.
