# Supplementary material for: Impact of Aspiration Pneumonia on the Clinical Course of Progressive Supranuclear Palsy: A Retrospective Cohort Study
Source: PLoS One. 2015 Aug 13;10(8):e0135823. doi: 10.1371/journal.pone.0135823 (PMC4536232; doi:10.1371/journal.pone.0135823)
Supplement: S3 Table — (DOCX) [file pone.0135823.s005.docx]

**S3 Table. Comparison of patients with RS and PSP-P**

|  | **All**  **(n=90)** | **RS**  **(n=48)** | **PSP-P**  **(n=13)** | **RS vs. PSP-P**  **(P value)** |
| --- | --- | --- | --- | --- |
| Age of disease onset, years (mean ± SD) | 68.6 ± 7.1 | 70.6 ± 6.6 | 67.7 ± 8.7 | 0.20^b^ |
| Male, n (%) | 58 (64) | 30 (63) | 9 (69) | 0.75^c^ |
| Cases with the experience of pneumonia, n (%) | 22 (24) | 10 (21) | 5 (38) | 0.28^c^ |
| Latency^a^ to dysphagia, years (mean ± SD) | 4.4 ± 2.7 | 3.1 ± 1.6 | 6.7 ± 3.4 | <0.001^b^ |
| Latency^a^ to first pneumonia, years (mean ± SD) | 7.9 ± 3.8 | 6.0 ± 3.3 | 10.4 ± 3.0 | 0.02^b^ |
| Case with NOF (non-oral feeding), n (%) | 21 (23) | 10 (21) | 4 (31) | 0.45^c^ |
| Number of deceased cases, n (%) | 16 (18) | 9 (19) | 4 (31) | 0.45^c^ |
| Total disease duration of deceased cases, years (mean ± SD) | 9.0 ± 4.6 | 6.8 ± 3.3 | 12.6 ± 6.2 | 0.048^b^ |
| Clinical features during the first 2 years of disease | |  |  |  |
| Fall episodes, n (%) | 60 (67) | 47 (98) | 2 (15) | <0.001^c^ |
| Cognitive decline, n (%) | 24 (27) | 23 (48) | 1 (8) | 0.01^c^ |
| Dysarthria, n (%) | 42 (47) | 27 (56) | 5 (38) | 0.35^c^ |
| Dysphagia, n (%) | 24 (27) | 20 (42) | 2 (15) | 0.11^c^ |
| Tremor, n (%) | 18 (20) | 7 (15) | 7 (54) | 0.006^c^ |
| Asymmetric onset of extrapyramidal signs, n (%) | 20 (23) | 7 (15) | 8 (62) | 0.002^c^ |
| Bradykinesia, n (%) | 52 (78) | 33 (77) | 10 (91) | 0.43^c^ |
| Postural reflex disturbance, n (%) | 52 (91) | 42 (95) | 3 (50) | 0.009^c^ |
| Extra axial-dystonia, n (%) | 25 (50) | 20 (49) | 3 (60) | 1.00^c^ |
| Supranuclear gaze palsy, n (%) | 35 (69) | 30 (75) | 2 (50) | 0.30^c^ |
| Abnormal saccade or pursuit, n (%) | 34 (76) | 31 (89) | 1 (25) | 0.01^c^ |
| Response to levodopa ever, n (%) | 21 (33) | 14 (39) | 3 (43) | 1.00^c^ |

^a^Latency from disease onset

^b^Mann-Whitney test

^c^Fisher’s exact test
